# Understanding the Role of Mobile Internet-Based Health Services on Patient Satisfaction and Word-of-Mouth

**DOI:** 10.3390/ijerph15091972

**Published:** 2018-09-10

**Authors:** Dongxiao Gu, Xuejie Yang, Xingguo Li, Hemant K. Jain, Changyong Liang

**Affiliations:** 1 The School of Management, Hefei University of Technology, Hefei 230009, China; gudongxiao@hfut.edu.cn (D.G.); xuejie_Y@126.com (X.Y.); lixingguo@hfut.edu.cn (X.L.); 2 The School of Informatics, Computing and Engineering, Bloomington, IN 47405-3907, USA; 3 College of Business, The University of Tennessee at Chattanooga, 615 McCallie Ave, Chattanooga, TN 37403, USA; hemant-jain@utc.edu

**Keywords:** patient satisfaction, mobile Internet-based health service, expectation confirmation model, patient participation, Internet medicine, patient word-of-mouth

## Abstract

With the rapid advancement of Web 2.0 technologies, Internet medicine, and mobile healthcare, the influence of the use of patient-oriented Mobile Internet-based Health Services (MIHS) on patient satisfaction and the electronic word-of-mouth (WOM) of health service agencies is becoming the focus of the academic research community. Many large hospitals, including some Internet hospitals, have provided various online healthcare service platforms that enable patients to expediently consult with physicians and obtain healthcare services in an online to offline format. The purpose of this study is to analyze the main mechanisms of how the features and users’ experiences of MIHS influenced patient satisfaction and continuous use behaviors of the system to generate additional WOM dissemination behaviors. Based on post-adoption behavior and Expectation Confirmation Model of Information Technology Continuance (ECM-IT), this study conducted an empirical study through data collection from users (patients) from a large hospital providing online healthcare services. A total of 494 pieces of data were collected and analyzed using SmartPLS2.0(SmartPLS GmbH, Hamburg, Gernmany). The results show that: (1) patient satisfaction with MIHS and their intentions to continue use of MIHS have significantly positive influences on WOM; (2) patient satisfaction with MIHS is positively influenced by perceived usefulness and confirmation of MIHS performance expectations; (3) and patient intentions to continue use of MIHS are also affected by some technology factors, such as facilitating conditions and perceived risk, as well as some subjective feelings, such as perceived usefulness and perceived interactivity. The results of this study provide important implications for both research and practice of public health.

## 1. Introduction

Health care is one type of service, which involves intangibility, heterogeneity and deep customer participation. With the advancement of Web 2.0 technologies, the traditional method of understanding disease and treatment has been changed, and an increasing number of public sector big hospitals are providing online healthcare service by using an online healthcare platform. Among these kinds of platforms, there are also a healthcare service and communication community in which patients can easily find preferred doctors and make appointments for diagnosis and treatment. For patients, finding a doctor through an online healthcare platform has significantly changed from the traditional method of finding a doctor in a brick-and-mortar hospital. In an online health community, patients can choose whether to make an appointment to see a doctor based on other patient word-of-mouth (WOM) of the hospital and doctors’ previous treatment results.

In practice, almost all healthcare service agencies expect patients to spread WOM online or offline, which helps them to earn a better reputation for attracting patients, as well as promoting mutual understanding between doctors and patients. In developing countries such as China, doctor-patient conflict issues are becoming one of hot topics for the entire society. The reasons for the conflicts are mainly due to distrust between doctors and patients, which are partly caused by information asymmetry and lack of effective communication. Some scholars focus on the topic of how to reduce information asymmetry and improve communication effectiveness between doctors and patients. Some studies examine how patients can improve their understanding of medical services provided by doctors and nurses in the hospitals from an information technology perspective. For example, Sundberg et al. tested the effect of the Information and Communication Technologies (ICT) platform on patient involvement and symptom assessment through efficient communication between doctors and patients [1]. Furthermore, some scholars discussed how the patient’s WOM reflect the medical service quality and doctor-patient’s trust [2,3]. Hence, from the social perspective, patient health is of great significance for enhancing the trust between doctors and patients, and thus improving patients’ WOM to health service agencies.

Prior research has paid attention to what affects consumer WOM behavior. It focused more on some factors, such as consumer satisfaction [4,5], emotion tendency [6], and some psychological factors—social interaction desire, economic incentives desire, and achieves self-worth, etc. [7,8]. In the field of medical research, some scholars have discussed the effect of WOM on patients’ or their agents’ decisions [9,10], and some studied the WOM from the view of patient interactions [11]. However, along with the rapid development of newly emerging technology such as mobile internet, social networking and interactive technologies, the era of internet medicine and mobile healthcare is forthcoming. Therefore, the increasing trend in patient-oriented Mobile Internet-based Health Service (MIHS) platforms have been widely used in practice. In MIHS, the WOM is influenced by some other new factors, such as doctor-patient interactions that affect the patient judgments of medical service quality, and influence their trusts. Hence, in the new platform of MIHS, it is necessary to further perfect the research on influence factors of WOM.

From the logic of forming WOM, patient satisfaction and their intentions to continue using MIHS are the main impacting factors. Both of them reflect the post-adoption states of patients after receiving treatments through an electronic platform. Patient satisfaction is related to patients’ subjective feelings. Their intentions to continue using MIHS are a subjective behavior. Prior research has proved the positive relationships between patient satisfaction and WOM [12]. The intentions to continue usage behaviors also reflect and strengthen the WOM [12]. Hence, WOM is influenced by patient satisfaction and their intentions to continue using MIHS.

Generally, a patient’s WOM is based on their perceived health care service quality. The prerequisite of WOM behavior occurrences is that patients are satisfied with the medical services of a hospital. Hence, a hospital that wishes to promote WOM should clearly know what factors affect patient satisfaction and how they enhance patient satisfaction. This is an important issue in examining patient WOM, which has been addressed by some scholars. For example, Jenkinson et al. summarized the major determinants of patient satisfaction and emphasized the effect of physical comfort, emotional support, and information communication, etc. [13,14]. However, patient satisfaction essentially refers to their subjective perception of MIHS. From the Expectation Confirmation Theory (ECT) perspective, the evaluation of satisfaction in the hospital is made by patients who compare their actual experience of receiving medical services from MIHS to the expectations of patients.

Although persistently applied in practice, the studies concerned with the role of MIHS on patient satisfaction and WOM have received little attention from scholars. In previous studies, researchers have primarily emphasized technology-based behavior and satisfaction rather than post-satisfaction behavior, such as continued use of services. For example, previous studies showed that a Hospital Information System (HIS) can promote patient satisfaction by improving doctors’ work efficiency, reducing waiting times, and offering patients convenient services and useful information [15]. Close relationships are also proven to exist in information technology along with the use behavior and outcomes [16,17]. Among these studies, various studies did not examine the patient post-satisfaction behavior. Additionally, the factors affecting patient WOM behaviors are diverse and complex, which likely include technical factors as well as social, economic, cultural and psychological factors. Moreover, MIHS has more convenient and interactive functions compared to traditional health service platforms. Few studies examine the role of MIHS on patient WOM. Thus, it is extremely important to explore factors affecting patient WOM and post-adoption behaviors in the context of Internet medicine, social networking, and interactive technology applications. To fill this gap, the current study draws upon the theory of post-adoption behavior and Expectation Confirmation Model of Information Technology Continuance (ECM-IT) to discuss the role of online health services on patient WOM to health service agencies.

On the basis of our related literature review, this research could be considered as first empirical study to examine MIHS’s role in promoting patient satisfaction, the continued use of MIHS and WOM. This study investigates the connections of factors for patient satisfaction, continued use and WOM. Therefore, this research work will be helpful for hospital management, offering a better understanding about the role of online healthcare service platforms to improve patient satisfaction and hospitals’ reputation. Moreover, it can also provide guidance for researchers and practitioners who design and develop these types of systems to support convenient and interactive online heath care services.

The rest of the paper is organized as follows: in Section 2, based on reviewing relevant theories and research, we develop a research model and propose hypotheses. Section 3 discusses the research methodology used to validate our proposed hypotheses. Section 4 presents the results. Finally, this paper concludes with a discussion of findings, implications for theory, practice and opportunities for future research in Section 5.

## 2. Research Model and Hypotheses Development

**Figure 1:**
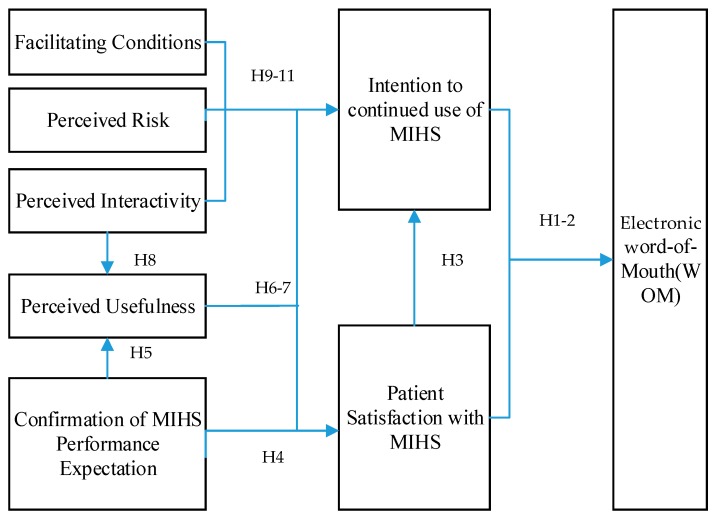
Research Model.

Drawing on ECT and Technology Adoption Model (TAM), Bhattacherjee proposed a model relevant to the continued usage of information technology (IT) based on ECT (ECM-IT) [18,19]. ECT emphasizes the positive association between confirmation and satisfaction, and states that both of these factors motivate people to repeat their behaviors [20]. ECM-IT focuses only on post-usage constructs, highlights the importance of post-usage expectations and uses perceived usefulness of post-usage to represent consumers’ post-adoption expectations, which differs from traditional ECT [19]. The main objective of this study is to examine the influence of online health service on WOM against the backdrop of Internet medicine. To achieve this goal, the Expectation Confirmation Model of Information Technology Continuance (ECM-IT) is utilized as the theoretical foundation. This study also incorporates facilitating conditions, perceived risk and perceived interactivity, which are the important factors affecting patient usage experiences with MIHS, and further influencing their intentions to continue use of MIHS. This could provide a more integrated model as shown in Figure 1.

### 2.1. Post-Adoption Behavior: Continuous Intention and WOM

Information systems (IS) continuance and WOM were suggested as the primary behavioral outcomes of the post-adoption stage [20]. IS continuance refers to an IS user’s decision to continue using a particular IS for a long period, which generally includes both continuance intentions and continuous use behavior [21]. In the IS research field, IS continuance is often used as a synonym for post-adoption behavior [22,23]. As another form of post-adoption behavior, WOM is defined as a channel for broadcasting product or service information [24,25,26,27,28]. Consumers often assess WOM information as having greater value than information in corporate brochures, because it is provided by friends, classmates or acquaintances rather than companies, and it is perceived to be more reliable [29,30]. Hence, WOM serves as a reliable source of information for customers [31]. It generally has a powerful influence on consumers’ assessment process on products or services, as well as subsequent decision behavior [28,32].

WOM has an important influence on customer behavior [33]. People often share their opinions in their social circles, such as with friends, classmates, relatives, etc. Prior marketing research has shown that a customer who is loyal due to his or her commitment to the product or service provider will recommend the same product or service to other customers [34]. The relationship between WOM and the repurchase intention has been validated in the research contexts of marketing, tourism or service management. Choo and Petrick found that WOM behavior is positively related to the repurchase intention. In the IS domain, few studies explored the relationship between WOM behavior and IS continued use intention [28,35]. In addition to previous studies, satisfaction is also considered as an important motivator of WOM behavior [36,37]. Satisfaction affects WOM behavior together with other stimuli that motivate WOM behavior, such as service quality, website quality, price perception of service, customer service strategies, loyalty, etc. [35,38]. Lam and Soan analyzed the survey data collected from tourists in Macao and revealed that satisfaction plays a crucial role in generating travel related WOM. Kitapci et al. identified the effect of satisfaction with WOM communication and repurchase intention, and found that satisfaction has a significant effect on WOM and repurchase intention [12]. Hence, our study argues that satisfaction and continued intention to use MIHS should be a prerequisite of WOM towards MIHS. The following hypothesis is proposed:

**Hypothesis 1** **(H1).** *A patient satisfaction with MIHS has a significantly positive effect on WOM towards MIHS.*

**Hypothesis 2** **(H2).** *A patient continued intention to use MIHS has a significantly positive effect on WOM towards MIHS.*

### 2.2. Expectation Confirmation Model of Information Technology Continuance (ECM-IT)

ECM-IT developed by Bhattacherjee has a solid theoretical foundation and it pays attention to the motivations for individual users’ IS continuance intentions that occur in the IS post-adoption stage [34]. Bhattacherjee found that the satisfaction of users affects the intention for continued use through prior use and perceived usefulness. In addition, previous empirical research supports the fact that users’ satisfaction is one major factor of) IT continued use [39,40]. In a study by Wong et al., performance expectancy has been proven to not only have a significant effect on use intention [41], but also on a user’s perceived usefulness [34]. User satisfaction is one salient factor that shapes continued use intentions [42]. Compared to prior studies based on a variety of theoretical perspectives such as TAM and Innovation Diffusion Theory that examines factors that cause users to initially adopt a new technology, ECM-IT addresses the factors which affect users to continue to use an IT after they had adopted the technology. Given lots of empirical support for the impact of continued use on IT success, it becomes critical to find the salient factors that affect post-adoption behavior of users that is either to continue or to discontinue use of a technology. ECM-IT is just a theoretical model developed specifically to understand continued IT usage behavior [43]. MIHS is designed to help patients to acquire information and provide feedback to hospitals [44].

The concept of patient satisfaction is derived from research associated with customer satisfaction. People evaluate their satisfaction by comparing the actual product or service with the expectation of the product or service before its purchase. The customers feel satisfaction with products or services if the perception is better than expectation. The research indicates that the use of IT has an effect on improving satisfaction of customers [45]. Individuals who choose to employ MIHS may have initial expectations, such as improving the efficiency of consultation and treatment, reducing time spending waiting in long lines, and communicating to hospitals their comments or suggestions associated with their personally experienced hospital management and health care services. In the Internet health care context, when a patient is satisfied with the MIHS, it is reasonable and rational to propose that he or she intends to continue specific types of internet healthcare services. Meanwhile, if a patient has used MIHS and his or her expectations have been confirmed, his or her impressions of the perceived usefulness of MIHS and satisfaction will be further enhanced. Moreover, MIHS has gradually penetrated the entire process of patients’ treatment in hospitals [46,47]. A patient can use MIHS in the processes of registrations, payment, consultations, proof of diagnosis and relevant inspection and report sheet checking and printing, treatment, post-operation health tracking and feedback, etc., and the platform has greatly improved the efficiency of hospitals [48,49]. When a patient thinks that the MIHS is useful for him or her to improve the efficiency of seeing a doctor or the accuracy of medical diagnosis, he or she will more likely be satisfied with MIHS and will continue to use it [50]. Thus, we hypothesize:

**Hypothesis 3** **(H3).** *A patient satisfaction with MIHS has a significantly positive effect on the continued intention to use MIHS.*

**Hypothesis 4** **(H4).** *A patient confirmation of MIHS performance expectations has a significantly positive effect on his or her satisfaction with MIHS.*

**Hypothesis 5** **(H5).** *A patient confirmation of MIHS performance expectations has a significantly positive effect on his or her perceived usefulness of MIHS.*

**Hypothesis 6** **(H6).** *A patient perceived usefulness of MIHS has a significantly positive effect on his or her satisfaction with MIHS.*

**Hypothesis 7** **(H7).** *A patient perceived usefulness of MIHS has a significantly positive effect on his or her intention to continued use of MIHS.*

### 2.3. Perceived Interactivity, Perceived Risk and Facilitating Conditions

#### 2.3.1. Perceived Interactivity

Interactivity is defined as “the extent to which users perceive their experiences as a simulation of interpersonal interaction and sense they are in the presence of a social other” [51]. Interactivity is a feature of technology, a process of message exchange, and a user’s perception after using a product or experiencing a service process [52,53]. Interactivity is supposed to be one of the key advantages of the Internet [54]. Previous studies investigated how interactivity affects users’ responses towards continuous intention and revealed that perceived interactivity is one of the determinants of continued use. Liu et al. found that interactive functions make individuals prefer to share and comment [55]. Additionally, prior studies also concerned the relationship between interactivity and usability. They found that perceived interactivity is one key determinant of perceived usefulness and user satisfaction [56]. Abdullah et al. found that consumer perception of hotel website interactivity influences the customer’s intention to revisit the website [57].

In this study, we focus more on the interactivity between an online healthcare service agency and patients because facilitating interaction between managerial or medical staff (doctors, nurses, etc.) is the design and implementation purpose of MIHS. For the remainder of this section, we will briefly review the theoretical relationship between perceived interactivity and continuous intention of use in the context of an interactive online system platform for medical services in hospitals. Interactivity is a predominant feature of MIHS [58]. MIHS allows patients to participate or engage more in the health care service process. Online messages, feedback, discussions and evaluation are main communication channels among patients and medical staff and commonly used to support the medical services of hospitals. 

In order to enhance our understanding of continued IT usage behavior, our study incorporates an additional user perception—perceived ease of use—into the original ECM-IT. Compared prior studies based on a variety of theoretical perspectives such as TAM and Innovation Diffusion Theory examining factors that cause users to initially adopt a new technology, ECM-IT addresses the factors which affect users to continue to use an IT after they had adopted the technology. Given lots of empirical support for the impact of continued use on IT success, finding the salient factors that affect post-adoption behavior of users, which is either to continue or to discontinue use of a technology, becomes critical. ECM-IT is just a theoretical model by developed specifically to understand continued IT usage behavior. Mobile Internet-based services is a new breed of IT innovation which is gradually becoming omnipresent in our daily life. Its usage encompasses a broad range of activities—both work-related activities and fun activities. Interactivity is an essential metric when evaluating MIHS. It includes not only interactions between hospital staff, doctors, nurses and patients, but also includes interactions or hyper linking between messages or comments [58] and uses plugins for links to share information. Because interactivity leads to jointly produced meaning or outcomes or reliance on judgments of credibility [58,59], the users of MIHS benefit, which will facilitate their perception of usefulness and motivate the intention of continuous use. While the usage of typical IT innovations in prior IS research are well defined, simple, and of limited function, the interaction of MIHS s is far more complex and comprehensive in supporting diversified health communication needs and medical care expectations. Hence, we hypothesize:

**Hypothesis 8** **(H8).** *Perceived interactivity has a significantly positive effect on the perceived usefulness of MIHS.*

**Hypothesis 9** **(H9).** *Perceived interactivity has a significantly positive effect on the continued use intention of MIHS.*

#### 2.3.2. Perceived Risk

The original concept of perceived risk proposed by Bauer [60] comes from psychology. Bauer defined perceived risk as “felt uncertainty regarding possible negative consequences of using a product or service” and believes that consumers’ purchase decisions are impacted by the uncertainty of the results [61]. When the level of uncertainty becomes more substantial, perceived risk will also increase [34]. Malhotra et al. found that risk is expected to exert a significant effect on behavioral intention [62]. Heijden et al. defined perceived risk as consumers’ subjective perception of negative consequences, and the probability that the unfavorable consequences may occur after the purchase of products [63]. Green and Pearson found that perceived risk reduction can increase continued behavior intentions [64].

Perceived risk has also been used to understand users’ post-adoption or resistance behavior toward usage of IT [65,66]. From the perspective of the patients, there are several types of risk involved with the use of MIHS systems. Compared to the general health service commonly used in the internal network in a hospital, MIHS is based on the Internet, and patients can access the platform anytime and anywhere. This means that MIHS will face more risks than traditional health services. Moreover, MIHS saves a large amount of private information, including the patients’ conditions, their concerns and questions, replies from the doctors, and patient opinions and comments regarding services from hospitals and doctors. These different aspects of risk are relevant in the continued use of MIHS. Thus, we hypothesize:

**Hypothesis 10** **(H10).** *Perceived risk has a significantly negative effect on the continued use of MIHS.*

#### 2.3.3. Facilitating Conditions

The Unified Theory of Acceptance and Use of Technology (UTAUT) [67] identified facilitating conditions as one of determinants that affect users’ behavior. Venkatesh et al. defined facilitating conditions as the degree to which a user believes that an organizational and technical infrastructure exists to support the use of information technology [68]. Hsieh et al. used the Theory of Planned Behavior (TPB) to identify several attitudinal, normative, and control beliefs that would predict continued IT use intentions. Hsish et al. found that facilitating conditions were particularly important among the underprivileged [69]. Venkatesh and Sykes also found that facilitating conditions and social network conditions existed, and they fostered the success of divided initiatives in developing countries [70]. Barnard et al. identified the powerful role that facilitated conditions have on digital technology use [71]. Liao et al. also found that facilitating conditions can seriously affect a person’s intention to use online services [72].

After the implementation of MIHS, many hospitals in developing countries such as China provided various facilitating conditions for patients to support the use of MIHS, which included making available: volunteers or support staff from the providers of software and service, self-service terminal machines, online information desks, operational instruction materials, operational procedure posters, WeChat access (WeChat is a new form of online connection created by Tencent, Inc., and with over 0.7 billion users from China, Europe, North America, and Southeast Asia and other counties or areas. It has become one of the largest and most widely used Internet portals worldwide; a hospital can collaborate with Tencent and open a WeChat Official Account (WeChatOA) for patients to access the online healthcare service platform via the hospital’s WeChatOA more conveniently and more quickly than traditional healthcare services.), etc. A user would obtain service support if something went wrong, and would then know how to proceed. All these resource and technology facilitating conditions allowed patients to feel comfortable, and helped them be able to manipulate MIHS or have sufficient learning opportunities to do so, and encouraged their continuous intention to use interactive information technology. Hence, we hypothesize:

**Hypothesis 11** **(H11).** *Facilitating conditions have a significantly positive effect on the continued use intention of MIHS.*

## 3. Research Methodology

### 3.1. MIHS Implementation and Use

**Figure 2:**
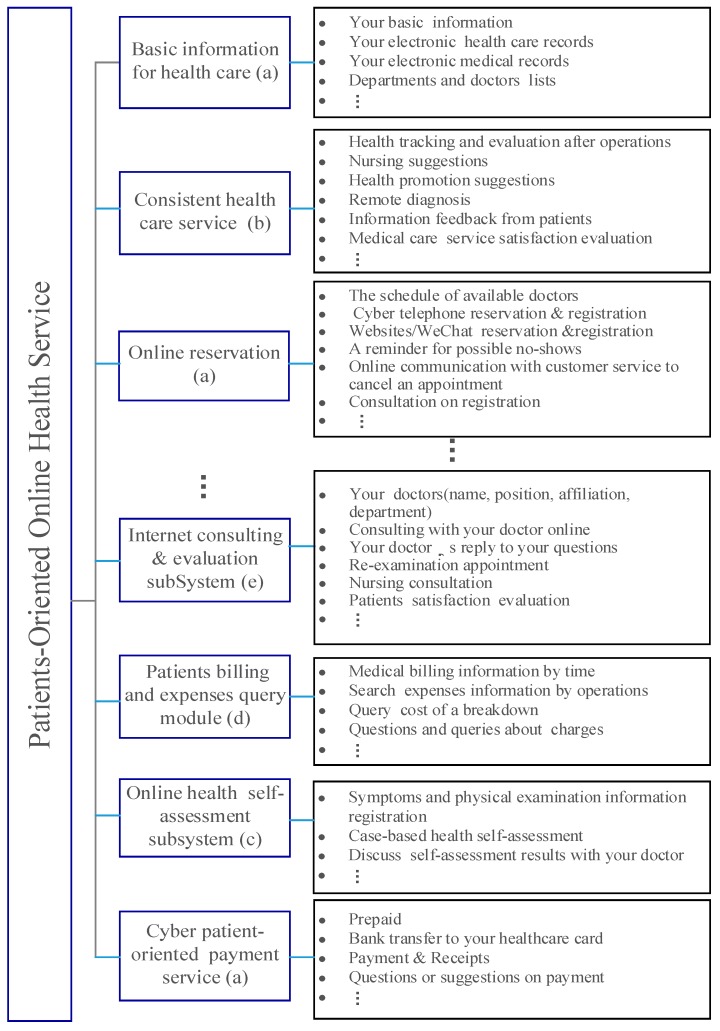
The main features of MIHS.

MIHS implementation began in 2013 in AN Hospital. Doctors and patients were tasked with promoting use of the system. Use of the system was rapidly popularized. The main functions of MIHS are summarized in Figure 2 in which we can find the main features of MIHS, which can benefit the patients as follows:

(a) Patients can access MIHS via the Internet or the hospital’s WeChat public account. Doctors’ information and their schedules are also available in the system, and patients can search, choose, and make appointments with their preferred doctors. They can complete registration, prepay online instead of waiting in line, and cancel appointments.
(b) The hospital can conduct post-operation health tracking and evaluation for a patient. The medical staff can obtain feedback from patients and offer medical and health-related suggestions, if necessary. Patients can communicate their symptoms to the doctors or nurses directly. They can discuss their current health status, postoperative rehabilitation, relevant precautions, etc. Additionally, the doctors can also provide online remote diagnosis services to patients with chronic diseases.
(c) Patients can assess their own health status based on a case-based health self-assessment subsystem. The type of assessment is based on historical cases and the whole life-cycle dynamic health data of the patients. Generally, only health status (level), but sometimes potential health risks and health promotion solutions can also be obtained. Patients can also consult with doctors about the assessment results and ask for suggestions.
(d) Patients can acquire various health care and expense information. Health care information includes electronic health records, physical examination reports, etc. Expense information includes registration fees, detailed operation fees, drug fees, etc. Digital health care reports can be printed out by patients via a procedure of application and verification. This allows patients to easily track care processing and outcomes. Patients can voice concerns about their treatment, the nursing process, and expenses.
(e) Patients can also perform medical care service satisfaction evaluations on doctors, nurses, departments, teams, the hospital, or a specific medical service event, such as an operation. They can leave detailed information about why and in what areas they are satisfied. The hospitals can conduct a satisfaction analysis based on the collected assessment data, which will be helpful for the promotion of its health care service.

### 3.2. Measures

Measures for all concerned variables were taken from previous studies and adapted to the context of healthcare. Eight variables were measured in this study: Confirmation of MIHS performance expectations, Perceived Usefulness (PU), Perceived Interactivity (PI), Facilitating Condition (FC), Perceived Risk (PR), Patient satisfaction with MIHS, Intention to continually use MIHS, and Electronic Word of Mouth (WOM). The questionnaires were formed by using Likert scales ranging from 1 (strongly disagree) to 7 (strongly agree), which requires respondents to select a number from the scale. In Table 1, the measurement items and their sources are listed.

To measure the confirmation of MIHS performance expectations, we used a four-item scale adapted from previous studies [73]. Facilitating Condition (FC) was measured by a three-item scale adapted from previous studies [74,75]. Intention to continued use of MIHS was measured by a two-item scale adapted from previous studies [74,76]. Perceived Interactivity (PI) was measured by a three-item scale adapted from previous studies [77]. Perceived Risk (PR) was measured by a three-item scale adapted from previous studies [78]. Perceived Usefulness (PU) was measured by a five-item scale adapted from previous studies [79,80]. Patient Satisfaction with MIHS was measured by a two-item scale and adapted from previous studies [80]. The seven-item scale for Electronic Word of Mouth (WOM) behavior was also adapted from previous studies [4,81,82,83,84]. The users of MIHS are patients and doctors, which are different from the classic users of a transactional application who use a system for their daily operations. To ensure that all measurement instruments are reliable and valid for our current study in the context of healthcare, we consulted with the relevant medical specialists and experts in the field of management information systems and conducted the necessary adjustments and adapted the scales to the special targeted respondents in the context of healthcare.

Based on these measures, we developed the survey questionnaire. After compiling the English version of the questionnaire, the items were translated into Chinese by a bilingual faculty member and then verified, refined, and back-translated for translation accuracy by a professor of healthcare information management. The content validity of all scales was established through both a literature review and a content validity expert panel comprising six faculty members and three doctoral students who are experienced in the research methods of quantitative and quantitative analysis. 

### 3.3. Sample and Data Collection

To validate the above research model, an empirical study was conducted at AN Hospital, a 3-A hospital and one of the largest hospitals in East China. The 3-A stratum represents some of the best hospitals in mainland China. This hospital has implemented patient-accessible MIHS, which allows it to provide mobile Internet-based healthcare services. Since we have a stable collaborative relationship with this hospital, our survey had full support from the hospital administrators. This allowed the data collection to be completed smoothly. A multistage iterative process was used for the data collection. First, we adapted the original measures from the literature (described in next section) into hospital context and then we translated the instrument into Chinese using professional translation staff. Second, we conducted a pilot study to improve ambiguous expressions, awkward wordings, or distortions of the original meanings using 100 respondents. Based on the data and respondents’ suggestions in the pilot study, we modified the questionnaire. The modified questionnaire was then used to collect data from patients at the hospital. The data collection lasted approximately two years, from July 2015 to June 2017.

Small gifts were given to the respondents for completing and returning the questionnaire. The gift with a value of approximately RMB 52.00 ($8.00). The questionnaires were randomly distributed to patients in the hospital. Patients who were not able to read or write were not selected to participate in the study. A total of 600 questionnaires were distributed and 528 questionnaires were returned; the response rate is 88%. After removing invalid questionnaires (incomplete, used same answers, obvious contradictions, etc.), we obtained 494 valid questionnaires with an effective response rate of 93.56%. Table 2 presents the demographic features of the respondents of this study.

## 4. Results

### 4.1. Measurement Model

Validation assessed the reliability of the measures, while hypothesis testing analyzed the hypotheses we proposed. Structural equation modeling with partial least squares (PLS) was used to perform a simultaneous evaluation of both the measurement quality (measurement model) and construct interrelationship (structural model). By using ordinary least squares as the estimation technique, PLS performed an iterative set of factor analyses, and applied a bootstrap approach to estimate the significance (*t*-values) of the paths [85,86]. Prior studies indicated that PLS-SEM overcame problematic model identification issues and that it is a powerful method for analyzing complex models using smaller samples [87]. Thus, in this study, we used SmartPLS2.0 to evaluate the measurement properties and test the hypotheses.

A latent variable is equal to λ* observed variable in which *λ* is a factor loading. The value of *λ* changes from zero to one and shows the correlation between observed variables and latent variables. Factor loadings in Table 3 present the means and loadings of each measured item and the descriptive statistics of each item. The loadings of all the items are above the threshold of 0.7, indicating that the observed variables have high convergent validity. The values in Table 3 also show a high correlation between observed variables and latent variables.

The acceptability of the measurement model was assessed by the reliability of the individual items, the internal consistency between the items, and the model’s convergent and discriminant validity. Table 4 shows the composite reliability, Average Variance Extracted (AVE), and square root of the AVE, as well as the correlations between the constructs. Scale reliability is an important measure of scale adequacy. When scale reliability is high, variables measuring a single factor share a high degree of common variance. The Cronbach’s alphas of the seven constructs are all above the recommended criterion of 0.70 [88] which shows that the measures are internally consistent. The composite reliability values of all the constructs are exceeding the cut-off value of 0.70 [89], which indicated adequate internal consistency [90]. The AVE for each construct is higher than 0.50, suggesting that the observed items explained more variance than the error terms [91]. In addition, the square root of the AVE for each construct was higher than the correlations between the construct and all other constructs, suggesting excellent discriminant validity. Thus, all scales of the measurement model demonstrate adequate internal consistency for further analysis of the construct model.

### 4.2. Common Methods Variance

To test for Common Methods Variance (CMV), we conducted Harman’s single factor test. According to Podsakoff et al., if a detrimental level of common method bias exists, “(a) a single factor will emerge from the exploratory factor analysis (unrotated) or (b) one general factor will account for the majority of the covariance among the measures” ([92], p. 889). In the exploratory factor analysis of this study, more than one factor emerged to explain the variance, and one general factor did not account for most of the covariance among the measures. Thus, the common method bias in this study is low.

### 4.3. Multicollinearity

To ensure that there is no risk of multicollinearity, we tested the data and found that none of the bivariate correlations was above 0.90 [93]. Additionally, the tolerance values, which are averaged to be greater than 0.30, are acceptable. The highest VIF among the constructs was 3.537. This is comparable to many prior studies and is well below the commonly accepted threshold: 10 [94]. This suggests that multicollinearity is not severe in our research model. To further demonstrate that our results are robust to multicollinearity, we removed the following measurement items (that caused high construct correlation) and estimated the model again: CPE01, CPE04, FC01, PU03, PI02, WOM06. As a result, all of the construct correlation values are below 0.75. The highest construct VIF is lowered to 2.521. Construct reliability and validity are still established. The results show that all of the path coefficients are significant at 5% level. Using multiple commonly accepted approaches, we have shown that our results are not threaten by multicollinearity.

### 4.4. Structural Model

**Figure 3:**
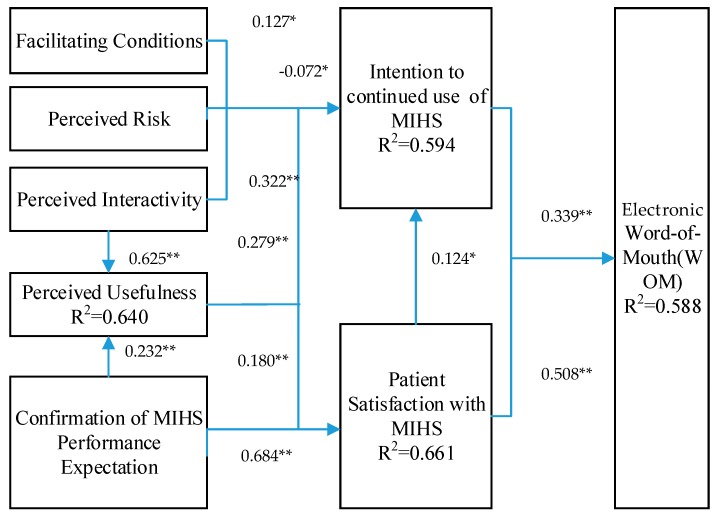
Model results. Path coefficients with *t* value in parentheses; * represents *p* < 0.05; ** represents *p* < 0.01.

To determine the statistical significance of the path coefficients, we ran the bootstrapping method setting the number of samples at 2000 and the number of cases at 494. The parameter estimated in the structural model exhibited the direct effects of one construct on the other; a significant coefficient at a certain level of *α* reveals a significant relationship between the latent constructs (Figure 3, Table 5).

H1, which hypothesized a positive relationship between patient satisfaction with MIHS and electronic Word of Mouth (WOM) behavior, was supported (path coefficients = 0.508, *p* < 0.01). Additionally, H2, which hypothesized a positive relationship between the intention to continue use of MIHS and WOM behavior, was also supported (path coefficients = 0.339, *p* < 0.01). Satisfaction with MIHS and the intention to continue use of MIHS explain 58.8% of the variance in WOM behavior. R^2^ represents the degree of interpretation of the dependent variable by the independent variable. 50%~60% is a suitable number. Patient satisfaction with MIHS has a positive and significant effect on Intention to continued use of MIHS (H3) with path coefficients of 0.124 (*p* < 0.05). As predicted by H4 and H5, confirmation of MIHS performance expectations significantly influenced patient satisfaction with MIHS and perceived usefulness with path coefficients of 0.684 (*p* < 0.01) and 0.232 (*p* < 0.01), respectively. As predicted by H6 and H7, confirmation of MIHS performance expectations and perceived usefulness significantly influenced the intention to continue use of MIHS with path coefficients of 0.18 (*p* < 0.01) and 0.279 (*p* < 0.01), respectively. Perceived interactivity had a positive and significant effect on perceived usefulness (H8) with path coefficients of 0.625 (*p* < 0.01). Perceived interactivity, perceived risk and facilitating conditions significantly influenced the intention to continue use of MIHS with path coefficients of 0.322 (*p* < 0.01), -0.072 (*p* < 0.05) and 0.127 (*p* < 0.05), respectively. The path coefficient of H10 is negative because we hypothesized that perceived risk had a negative impact on the continued use intention, that is, the higher the perceived risk, the less likely the patient would continue to use MIHS. All these indicators showed that the model fit the data well. We will discuss these findings in detail in the next section.

## 5. Discussion, Implications and Limitations

### 5.1. Discussion

In this study, we focused on the impact factors that influence patient electronic word-of-mouth (WOM) based on post-adoption behavior and ECM-IT. The empirical results supported all research hypotheses and proved the significant impact of the use of MIHS on patient WOM behaviors. The supported H1 and H2 indicated that patient satisfaction and the continuous use of MIHS had a significant positive influence on improving the patient WOM, and the supported H3 shows that satisfaction with MIHS could promote the intention to continue using MIHS. The empirical results of H6 and H7 indicated that the perceived usefulness of MIHS had a positive influence on patient satisfaction and continuous use of MIHS. Both the perceived interactivity and confirmation of MIHS performance expectations positively and significantly affected the perceived usefulness.

From the IT perspective, we mainly studied MIHS. MIHS is an emerging generation of healthcare system platforms that provide interactions between hospital staff and patients, and allow patients to access various pieces of healthcare information through smartphones. The main characteristics of MIHS are accessible to both doctors and patients, and allows for convenient interactions with one another. The use of this novel MIHS is powerful for doctor-patient interactivity experience.

In our study, the perceived interactivity enhanced the perceived usefulness. This is understandable because the interactivity of MIHS helps patients ascertain their healthcare situation instantly and obtain suggestions by consulting with their doctors at any time. Perceived interactivity and perceived usefulness helped users make decisions on their continued use of MIHS. Perceived usefulness also promoted patient satisfaction experience. The patients with higher satisfaction levels were more likely to continuously use MIHS, which was consistent with a previous study [16]. The patient expectation confirmation on MIHS performance was also an important factor when evaluating the patient perceived usefulness of and satisfaction with the system. Facilitating conditions, such as increasingly convenient WiFi and 3G networks, as well as increasingly widespread use of smartphones and WeChat, further enhanced continued use of MIHS. The reduction of perceived risk also improved users’ intention to continued use of MIHS, further motivating their WOM behavior.

There are five pathways to “use intention” (H9 vs. H8 + H7). The pathways are a little complicated but is normal in this kind of SEM-based behavioral and empirical study. Based on the results of data analysis, there are two main pathways (path coefficients are over 0.25). For a specific individual, it is possible for him to have high perceived interactivity but low perceived usefulness. But for our study, our conclusion is based on SEM-based statistical analysis. This phenomenon with high perceived interactivity but low perceived usefulness will not cause influence on our conclusion.

### 5.2. Implications

#### 5.2.1. Implications for Research

The main purpose of our research was to explain the mechanisms of how the features of MIHS and users’ experiences affect patient satisfaction and their intention to continue their use of the platform, eventually spreading WOM. This research may have some implications for academic studies. First, our study extended the post-adoption behavior and ECM-IT to the context of mobile Internet medicine. MIHS, the object of our study, is a new type of mobile health service that can be used for knowledge discovery based on electronic medical records and health examination reports; doctors and nurses can use this system for timely communication and information feedback. As a new type of interactive internet medicine and mobile health service using data mining technologies, it greatly improves the efficiency and communication convenience of clinic services, which is completely different from the traditional and relatively closed HIS system. We examined the benefits of using MIHS in improving patient satisfaction, continued use, and eventually WOM. As users of MIHS, the patients were different from employees who used information systems in the business field. Thus, our focus was different from the IS post-adoption in the traditional organizational context that has been examined by other researchers, making this study a unique contribution.

Second, based on ECM-IT, our study developed a new model by adding perceived interactivity as mediated instruments. Prior studies investigated perceived usefulness and perceived ease of use in influencing users’ IT behavior [16,17]. IS users’ continuous use behaviors depend on not only the features of technology itself but also the users’ experiences, including perceived interactivity and confirmation of IS performance expectations [2,3]. Scholars who engaged in studies associated with post-adoption behavior seldom addressed users’ experiences during the technology use process. Especially in the scope of internet medicine and mobile healthcare services, the continuous use behaviors of online healthcare service toolkits are more closely related to perceived interactivity and technology performance expectation confirmation [95].

In addition, another contribution of this study is to provide a new perspective for other scholars to theoretically investigate the influencing mechanism of healthcare information technology on user experiences, managerial effectiveness, and organizational reputation. We explored the positive impact of patient satisfaction and continued use intention of MIHS on WOM, which could further influence the trust and relationships between doctors and patients [96]. In fact, the purposes of IT use are to improve efficiency and performance. In the context of online healthcare, the continued use of MIHS could improve the communication efficiency between doctors and patients, encourage trust [97], and improve the WOM of patients. Our research could help promote this type of study in theory to further focus on the purpose and effectiveness of IT implementation in depth in the internet medicine era.

#### 5.2.2. Implications for Practice

This research could also benefit hospitals and other healthcare providers. This research could encourage hospital decision-makers to pay attention to how MIHS plays a role beyond its deployment and focus more on the impact of the users’ expectations, perceived risk, perceived usefulness and perceived interactivity on MIHS use. WOM is an important source of trust between doctors and patients and plays a significant role in improving the relationship between them. MIHS is an innovative health service in terms of patient satisfaction. According to its positive effect on WOM in our empirical tests, hospital decision-makers should pay more attention to the continuous and effective operation of MIHS. Some key factors are discussed in our studies, including technology features and user experience, which would influence the satisfaction and continuous operation of MIHS.

First, from the perspective of technology features, the hospitals should strengthen the facilitating conditions and safety management of MIHS. As multiple terminals and the interactivity of information management system are involved, risks such as information leakage and attack will affect users. Therefore, efficient safety management could reduce the risks in the system and improve patient’s trust and perceived reliability. Moreover, the operation of MIHS also must be simplified to improve perceived ease of use and improve their satisfaction.

Second, from the perspective of user experience, our studies have shown that both perceived usefulness and perceived ease of use have a positive influence on the continuous use of MIHS and the improvement of patient satisfaction. These results are similar to the results of Ong, Lai and Wang’s research in the context of e-learning systems [98]. To improve continuous use, the basic function of MIHS should be enriched to improve perceived usefulness. In addition, hospital managers should enhance the interactivity functions of MIHS, which are key factors influencing the patient perceived usefulness and intention for continuous use. This includes ensuring system stability and continuous openness, guaranteeing sufficient interactive time between medical staff and patients, and so on.

### 5.3. Limitations

This paper analyzed the main mechanisms of how the features and users’ experiences of MIHS influenced patient satisfaction and continuous use behaviors of the system to generate additional WOM dissemination behaviors. This study could link the improving trust of doctors and patients to the use of MIHS. However, there are some limitations that can be addressed by further studies.

Firstly, we used continued use intention rather than actual use behavior. In the research area of information systems use, most researchers only studied behavioral intention rather than behaviors [99], because it is not easy to obtain the real use data by survey methodology. We did not follow up and track the actual use of MIHS, considering its difficulty and complications. It is a challenging issue to explore other means of obtaining data. It is a further challenge to include other research methods to obtain data indicating specific individual use of modules from MIHS data bases, as permitted by policies associated with the use, disclosure, and privacy protection of individual healthcare data. Future studies could consider how to establish dynamic tracking mechanism of patients continued use behaviors.

Secondly, this paper did not analyze individual differences, so the impact mechanisms of MIHS on patient satisfaction in different populations are not clear. In fact, the attribute variables have shown a significant influence in the medical service area [100,101]. Future studies should further explore the moderating effects of these attribute variables.

The third one is that our study did not consider excluding patients who could not read or write although the number of such participants is small.

## 6. Conclusions and Future Directions

Based on post-adoption behavior and ECM-IT [102], this research constructs a comprehensive model to explain the mechanisms of how the features of MIHS and users’ experiences affect the output of MIHS use, such as patient satisfaction, intentions of continued use and WOM. Patient satisfaction, intentions of continued use and WOM are the key factors influencing the relationship between hospitals, doctors and patients. Based on the data from patients at AN Hospital, one of the largest hospitals in East China, we tested the model.

The relationship between doctors and patients has become increasingly important and sensitive, which could influence patient satisfaction of medical services. Some hospitals have attempted to introduce the MIHS to improve their service quality. However, there are two questions that need to be answered. First, how is the MIHS used to improve patient satisfaction and how does it stimulate its continuous use. Second, what is the effect of MIHS use on three outputs: patient satisfaction, continued use of MIHS, and WOM.

In this study, we established a research model on the impact of using MIHS on patient WOM from the perspective of both technological features and users’ experience, and we empirically tested the model with data collected from AN Hospital. All the research hypotheses were supported. According to the above analysis and discussion, we found that the facilitating conditions of MIHS technology had a positive impact on the patients’ intention to continue using MIHS, and the relationship between perceived risk and continual use of MIHS is negative. In addition, perceived interactivity and conformity with the patients’ MIHS performance expectations also influenced patient perceived usefulness, thus improving patient satisfaction and stimulating their continuous use behaviors. The above analysis could answer the first question about the mechanism of MIHS impact. We further discussed the second question about the effects of using MIHS. The results showed a significantly positive influence of using MIHS on patient WOM behaviors. That could promote trust between hospitals or doctors and patients, and improve their relationships. Our research has a role in the expansion of ECT theory and a guiding role in the practice of mobile Internet-based healthcare service. Meanwhile, there are some possible directions for the future as follows.

The first one is that the depth difference of MIHS use could exist. Most of the participants used a majority of the functions of interactivity such as consultation on registration, satisfaction evaluation, health situation consultation with medical staff, bills consultations, communication with other users and etc. In our study, we consider different types of interaction with MIHS but failed to successfully communicate with the hospital and get the data of interaction in detailed granularity. This is an interesting issue deserving further research in the future.

Secondly, in our study, we did not investigate the pre-variables (such as cost-of-care, health outcomes, etc.) of patient expectations, usefulness, and perceived risk. The issue associated with these factors and corresponding influential mechanism is really very interesting and deserves further study in the future.

Last but not least, researchers can extend our research to the perspective of the role of IT use on patient satisfaction and WOM of medical service. In our study, the research objective is MIHS, not medical service of the hospital in which cost-of-care and health outcomes can affect patient expectations, usefulness, and perceived risk. This issue associated with the role of MIHS use and medical care output will be more interesting and challenging.”

## Figures and Tables

**Table 1:** Measures of constructs.

Construct	Item ID	Items	Reference
Confirmation of MIHS performance expectations	CPE01	My experience with using MIHS was better than what I expected.	Hong et al., 2006 [73]
	CPE02	The service level provided by MIHS was better than what I expected.	
	CPE03	The service level provided by MIHS are really same as what I expected.	
	CPE04	Overall, most of my expectations from using MIHS were confirmed.	
Facilitating Conditions	FC01	I have the resources necessary to use the MIHS system.	Ajzen 1991 [74]; Taylor and Todd 1995 [75]
	FC02	I have the knowledge necessary to use the MIHS system.	
	FC03	Given the resources, opportunities and knowledge it takes to use the MIHS system, it would be easy for me to use the system.	
Intention to continued use of MIHS	ICU01	I intend to continue using MIHS frequently during the next three months.	Ajzen 1991 [74]; Ajzen and Madden 1986 [76]
	ICU02	I intend to continue using MIHS for online registration, bills checking, consultation, evaluations, and etc. during the next three months.	
Perceived interactivity	PI01	MIHS allows me to interact with it to receive various health service or communicate with others.	Song and Zahedi, 2005 [77]
	PI02	MIHS has interactive features, which help me accomplish my task.	
	PI03	I can interact with the MIHS system in order to get specific information or provide feedback/evaluation.	
Perceived risk	PR01	I think it is risky to provide my personal information in the MIHS system.	McKnight, et al., 2002 [78]
	PR02	I think it is risky to input my bank card information for registration, bill paying or prepaid.	
	PR03	Entering personal information over the MIHS system is unsafe	
Perceived usefulness	PU01	The MIHS system is useful for searching and obtain the information I need.	Yoon, 2009 [79]; Bhattacherjee & Premkumar, 2004 [80]
	PU02	The MIHS system enhances my effectiveness in obtaining healthcare service.	
	PU03	The MIHS system enables me to get healthcare service faster.	
	PU04	Using MIHS system will improve my performance.	
	PU05	Using MIHS system will increase my productivity during health service process.	
Patient Satisfaction with MIHS	PS01	I am pleased with my use of MIHS system.	Bhattacherjee & Premkumar, 2004 [80]
	PS02	I am satisfied with my use of MIHS system.	
Electronic word-of-mouth (WOM)	WOM01	I am willingness to recommend MIHS to others.	Harrison-Walker, 2001 [81]; Anderson, 1998 [4]; Andrei, 2013 [82]; Kim, et al., 2001 [83]; Singh, 1990 [84]
	WOM02	Exactly I will tell the other person that MIHS is very good.	
	WOM03	I am willing to tell other people about the good aspects of MIHS.	
	WOM04	Told my friends and relatives about my good experience of using MIHS.	
	WOM05	I will mention this good service of MIHS to others quite frequently.	
	WOM06	I will tell more people about the service of MIHS.	
	WOM07	I am proud to tell others that I use MIHS service.	

MIHS: patient-oriented Mobile Internet-based Health Services.

**Table 2:** Sample Demographics.

Category	Number (%)
**Gender**	
Male	253 (51.21%)
Female	241 (48.79%)
**Age**	
<18 years old	4 (0.81%)
18–28 years old	103 (20.85%)
28–48 years old	169 (34.21%)
48–60 years old	103 (20.85%)
>60 years old	115 (23.28%)
**Educational background**	
Elementary school	62 (12.55%)
Middle school	123 (24.90%)
High school	135 (27.33%)
College	162 (32.79%)
Graduate school	12 (02.43%)
**Types of interaction, use duration with MIHS (multi-choices)**	
Consultations about registration, card opening, prepay	456 (92.31%)
Consultation about bills (avg. use duration of this function: 23.4 months)	471 (95.34%)
Satisfaction Evaluations (avg. use duration of this function: 20.7 months)	429 (86.84%)
Consulting individual diseases with medical staff (avg. use duration of this function: 19.9 months)	415 (84.01%)
Others such as appointment cancelling, nursing consultation, etc. (avg. use duration of this function: 21.3 months)	436 (87.85%)
**Treatment duration days**	
Outpatient	32 (06.48%)
Inpatient <5 days	96 (19.43%)
Inpatient 6–10 days	156 (31.58%)
Inpatient 11–20 days	133 (26.92%)
Inpatient >20 days	77 (15.59%)

**Table 3:** Descriptive statistics of the measure.

Construct	Item Statistics			
	Construct Items	Mean	Std. Deviation	Loading ^1^
Confirmation of MIHS performance expectations	CPE01	5.60	1.23	0.7951
	CPE02	5.44	1.24	0.8270
	CPE03	5.61	1.23	0.8508
	CPE04	5.78	1.19	0.8730
Facilitating conditions	FC01	5.46	1.49	0.7480
	FC02	5.65	1.38	0.8544
	FC03	5.69	1.38	0.8572
Intention to continued use of MIHS	ICU01	5.69	1.20	0.8950
	ICU02	5.62	1.29	0.8848
Perceived interactivity	PI01	5.77	1.20	0.8293
	PI02	5.86	1.19	0.8662
	PI03	5.65	1.28	0.8163
Perceived risk	PR01	3.41	1.93	0.8708
	PR02	4.12	2.04	0.8440
	PR03	3.86	1.91	0.7674
Perceived usefulness	PU01	6.07	1.10	0.8150
	PU02	6.02	1.10	0.7965
	PU03	6.02	1.05	0.8435
	PU04	5.93	1.16	0.8177
	PU05	5.83	1.18	0.7494
Patient satisfaction with MIHS	PS01	5.75	1.45	0.8950
	PS02	5.63	1.46	0.8848
Electronic word-of-mouth(WOM)	WOM01	5.99	1.18	0.7999
	WOM02	6.17	1.06	0.7809
	WOM03	6.04	1.09	0.8258
	WOM04	5.71	1.23	0.7823
	WOM05	5.88	1.17	0.8102
	WOM06	6.06	1.07	0.8084
	WOM07	5.74	1.20	0.7869

^1^ The loading is reported by SmartPLS 2.0. It shows a high correlation level between observed variables and structural variables.

**Table 4:** Measurement model results.

	Composite Reliability	Cronbach’s Alpha	AVE 1	CPE	FC	ICU	PI	PR	PU	PS	WOM
**CPE**	0.8812	0.7981	0.7121	**0.8439**							
**FC**	0.8705	0.7769	0.6916	0.7992	**0.8316**						
**ICU**	0.8839	0.7374	0.7919	0.6429	0.6063	**0.8899**					
**PI**	0.8756	0.788	0.7013	0.677	0.6183	0.7159	**0.8374**				
**PR**	0.8676	0.7812	0.6865	−0.2154	−0.2115	−0.2218	−0.2235	**0.8286**			
**PU**	0.8995	0.851	0.6912	0.6546	0.5965	0.6918	0.7817	−0.1018	**0.8314**		
**PS**	0.9003	0.7786	0.8187	0.8016	0.7924	0.6238	0.6539	−0.1842	0.6277	**0.9048**	
**WOM**	0.9294	0.9134	0.6221	0.7336	0.6848	0.6561	0.7174	−0.1365	0.7887	0.7199	**0.7887**

^1^ AVE stands for Average Variance Extract. The bold numbers listed diagonally are the square root of the variance shared between the constructs and their measures. The off-diagonal elements are the correlations among the constructs. For discriminate validity, the diagonal elements should be larger than the off-diagonal elements.

**Table 5:** Structural parameter estimates.

Hypothesized Path	*t*-Value	Results
H1: Patient satisfaction with MIHS → WOM	13.244 **	Supported
H2: Intention to continued use of MIHS → WOM	8.194 **	Supported
H3: Patient satisfaction with MIHS → Intention to continue use of MIHS	2.041 *	Supported
H4: Confirmation of MIHS performance expectation → Patient satisfaction with MIHS	20.086 **	Supported
H5: Confirmation of MIHS performance expectation → Perceived usefulness	5.021 **	Supported
H6: Perceived usefulness → Patient satisfaction with MIHS	4.606 **	Supported
H7: Perceived usefulness → Intention to continued use of MIHS	4.861 **	Supported
H8: Perceived interactivity → Perceived usefulness	13.77 **	Supported
H9: Perceived interactivity → Intention to continued use of MIHS	4.799 **	Supported
H10: Perceived risk → Intention to continued use of MIHS	2.484 *	Supported
H11: Facilitation conditions → Intention to continued use of MIHS	2.166 *	Supported

* represents *p* < 0.05; ** represents *p* < 0.01.
